# Opportunities and challenges for family-centered postpartum care during the COVID-19 pandemic: a qualitative study of nurse perspectives

**DOI:** 10.1186/s12912-022-00875-5

**Published:** 2022-04-27

**Authors:** Katharine W. Buek, Molly O’Neil, Dorothy J. Mandell

**Affiliations:** Population Health, Office of Health Affairs, University of Texas System, University of Texas Health Science Center at Tyler, 210 W. 7th St, Austin, TX 78701 USA

**Keywords:** Postpartum nursing, COVID-19, Parent education, Family-centered care

## Abstract

**Background:**

The global COVID-19 pandemic has forced the health care sector to make wide-ranging changes to protect patients as well as providers from the risk of infection. Many of these changes are likely to have greatest impact in contexts of care that employ family-centered care (FCC) models, including perinatal and maternity care. Research conducted in perinatal care settings during the pandemic has shown that some of these restrictions have negatively impacted patient and family experiences and outcomes, while others have been perceived as beneficial. The present qualitative study aimed to understand what changes have occurred in postpartum nursing practice during the pandemic, and how these changes have affected nurses, women and families during their stay in the hospital following a new birth.

**Methods:**

Structured interviews were completed with 20 postpartum nurses from five hospitals across Texas. The interview protocol was designed to elicit information about changes to hospital policies in postpartum units during the pandemic, nurses’ attitudes about these changes, perceived benefits and challenges for performance of their duties, and perceived effects on patients and their families. Nurses were recruited for the study using a purposive sampling approach. Interviews were conducted by video conference using Zoom and lasted approximately 30 to 45 min. Data were analyzed using a qualitative descriptive approach.

**Results:**

Participants reported that their hospitals placed restrictions on the number and mobility of support persons allowed to stay with the mother in the unit and prohibited all other visitation. Some challenges of these policies included reduced opportunities for hands-on learning and an increased number of patients opting for early discharge. Perceived benefits for patient education and outcomes included improved frequency and effectiveness of nurse-family communication, increased father involvement, and greater opportunities for maternal rest, breastfeeding, skin-to-skin care and family bonding.

**Conclusions:**

Study findings suggest that some limitations on postpartum hospital visitation may achieve important, family-centered goals. Protected time for family-bonding, maternal rest, breastfeeding, father involvement and individualized education are critical to quality FCC. Research must examine which visitation policies maximize these benefits while preserving patient access to family and social support.

## Introduction

Since the outset of the global COVID-19 pandemic, the health care sector has been forced to make wide-ranging changes to meet the threefold challenge of providing quality health care to patients, reducing the spread of the virus among the population, and protecting the frontline workforce. In-person visits have been curtailed or replaced by virtual options where possible. When in-person care is required, precautions have included restrictions on the participation of visitors and support persons, use of personal protective equipment (PPE), and social distancing or isolation of patients from each other and from providers, support persons, and visitors. These restrictions are likely to have greatest impact in contexts that employ family-centered models of care, which emphasize the inclusion and involvement of parents, relatives, and extended social support networks to the extent desired by patients and families [1–3]. It is important to understand how changes in health care related to the COVID-19 pandemic may have affected the practice of family-centered care (FCC) for women and families during this period.

### Family-centered postpartum care

The involvement of family and other sources of social support in the perinatal period has been associated with reduced pregnancy risk factors, lower maternal stress and depression, and improved birth outcomes [4–7]. The foundational principles for family-centered postpartum and newborn care include: 1) parents have access to the infant and are able to participate in all aspects of newborn care, 2) patient and family autonomy and authority are respected (above and beyond the convenience of healthcare providers), 3) patients have access to family and social support to the extent desired, and 4) healthcare providers give individually tailored education designed to empower parents during their transition into the parenting role [8–10]. Research has identified certain characteristics of high-quality postpartum education that increase parent confidence and reduce anxiety upon discharge from the hospital. Buchko, Gutshall and Jordon [11] and Weiss and Lokken [12] note that high quality postpartum education should:

- Be tailored to parents’ unique needs and interests
- Include the support person(s)
- Be provided in small amounts over the course of the hospital stay
- Be provided at times that are convenient for the patient and support person
- Include opportunities for hands-on practice

Alternatively, Ellberg, Högberg and Lindh [13] found that some of the most significant sources of parents’ dissatisfaction with postpartum care included a lack of respect or acknowledgement of the father in interactions with hospital staff; a lack of sensitivity, support and attention from overworked and stressed-out nursing staff; and a lack of information and preparation for the transition to life at home.

### Postpartum care during COVID-19

Studies investigating changes in perinatal care during COVID-19 have identified negative outcomes associated with hospital policies barring the presence of a support person from labor and delivery [14], separating mothers from their infants during the postpartum period [15, 16], and restrictions on parental visitation in the NICU [17]. Some COVID-related changes have compromised patients’ physical and mental health [18]. Bradfield et al. [19] explored the experiences of women, support persons, and providers of maternity care during COVID-19 in Australia. Healthcare providers, including midwives, medical professionals and midwifery students experienced stress and feelings of isolation from their patients, but generally held more positive views of the care they were providing than did patients and families (about the care they received). Partners or other support persons, in particular, described their experience as “stressful,” “disappointing,” “confusing,” and reported feeling “isolated,” “excluded,” and “sad.” In another Australian study, Vasilevski et al. [20] found that support persons experienced significant stress during the postpartum visit, citing the trauma of being separated from the mother and child immediately after the birth, the extra pressure of being the sole support person allowed, and a lack of support for their emotional and psychological well-being. The study also found that women and support persons perceived certain benefits to restricted visitation, including more time to rest and bond with the baby.

### Study purpose

At the time of writing there were no studies examining changes in patient and family education and care in postpartum contexts during COVID-19. Postpartum nurses are the frontline of family-centered postpartum care. They have the most direct contact with the mother, baby and support person immediately following the birth and are responsible for providing acute care as well as education to assist the new family in the transition from hospital to home. During the 2- to 4-day postpartum stay, nurses provide critical information on infant safety, breastfeeding support, and maternal care. Nurses also facilitate parent-infant bonding and provide basic instruction in infant care to new parents. It is important to understand how changes due to COVID-19 may have affected these key functions. The present qualitative study aimed to understand what changes have occurred in postpartum nursing practice during the pandemic, and how these changes have potentially impacted the provision of postpartum care and parent education, the inclusion of support persons and other family, and nurse-family interaction during the postpartum hospital stay.

## Methods

### Protocol development

This analysis utilizes data that was collected as part of a larger study of nurse attitudes and practices regarding father involvement in postpartum education and newborn care. Some of the interviews for this larger study took place in late 2020 and early 2021, several months into the COVID-19 pandemic. By this time, hospitals had imposed several iterations of pandemic-related policies and restrictions, which changed as more information about the virus became available and as its prevalence and severity fluctuated in local populations. This study focuses on the questions specific to COVID-19 policies and practices in the interview protocol. These seven questions captured information about changes in hospital policies due to the pandemic, nurse perceptions of the impacts of these changes on their ability to provide care, and nurse perspectives on patient and family response to the changes.

Data collection protocols were approved by the University of Texas Health Center at Tyler institutional review board (IRB) and a waiver of written consent was granted. Information about the study was provided to participants and verbal consent was obtained prior to the start of interviews. All research activities were conducted in accordance with IRB guidelines and requirements.

### Participants

Nurses were recruited for the study using a purposive sampling approach to represent as many different hospitals as possible. Study coordinators distributed information about the purpose and parameters of the study via email to colleagues with established relationships with postpartum units in hospitals throughout the state of Texas. Nurses who were interested in participating in the study were asked to contact the study coordinators. To be eligible for participation, nurses had to have current or recent experience working in the postpartum unit, preferably in a position involving postpartum rounding (daily visits with patients and their families). Participants were asked to refer colleagues who would like to participate in the study.

Interview participants included 20 female postpartum nurses working in five hospitals located in cities in the Gulf Coast, Central, Northern and Panhandle regions of Texas. At the time of data collection, 19 nurses had at least 1 year of experience working in postpartum care pre-pandemic; one had started working in postpartum care shortly after the start of the pandemic in spring 2020. One participant was a parent educator with prior experience as a postpartum nurse.

### Data collection and analysis

The first author, an experienced researcher with doctoral level training in qualitative research methods, conducted all interviews by video conference using Zoom (Zoom Video Communications), each lasting approximately 30 to 45 min. Interviews were audio-recorded with participants’ permission. Written consent was waived for this study, because the signed consent form would have been the only documentation linking participants with their data. Interview results were not shared with hospitals where nurses worked, however, it was believed that nurses would be more forthcoming about the impacts of hospital policies if their responses were anonymous. Verbal consent was obtained prior to the start of the interviews. Upon completion of the interviews, participants received a $75 electronic gift card as compensation for their time. Audio recordings were transcribed using Rev.com, a reputable online transcription service.

This study employed a qualitative descriptive (QD) approach [21, 22]. QD is a research design or framework that qualifies the overall purpose and approach to the research question, which is to simply describe phenomena as they are presented by the research participants. Researchers using a QD approach “stay closer to their data and to the surface of words and events than researchers conducting grounded theory, phenomenologic, ethnographic, or narrative studies” ([22], p. 336). In this case, the purpose of the study was to describe nurses’ attitudes and experience during COVID-19.

**Fig. 1:**
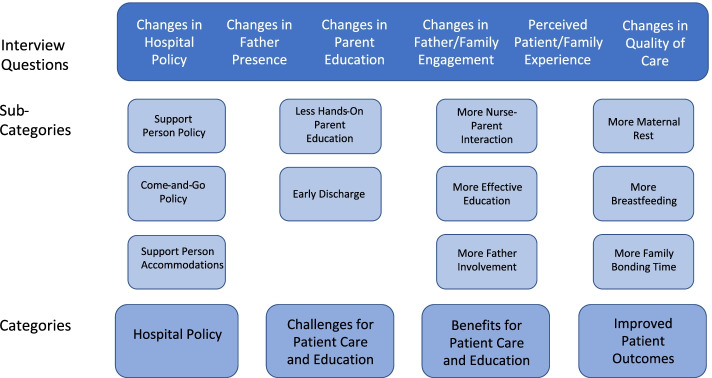
Final coding scheme resulting from content analysis

The data were analyzed using content analysis [21–23]. First, open codes were generated within the interview transcripts using an inductive approach. These were then grouped according to topics (or sub-categories), and then into higher-order categories. For instance, participants’ responses to the question “What changes has your hospital instituted with regard to the COVID pandemic...?” typically included information about who was allowed to attend the mother during her postpartum stay and rules regarding whether and when patients or support persons could leave the room or hospital. Open codes were created for these responses, including for example “one support person only, “one support person per day,” and “leave, but no return.” These codes were then grouped into sub-categories, for example: “support person policy” and “come-and-go policy.” Finally, sub-categories were grouped into higher-order categories (e.g., “hospital policy”). The final coding structure is shown in Fig. 1, below.

Data coding and analysis were done in NVivo Version 12 [24]. Coding was conducted by two of the authors. All ambiguous cases were discussed to reach consensus. Coders also reviewed 10% of each other’s coded transcripts to check for agreement. No discrepancies were encountered. After 20 interviews, saturation was reached for all categories and sub-categories.

## Results

### Hospital policy

The nurses in this study reported that their hospitals placed restrictions on the number of support persons allowed in postpartum units and the degree to which support persons could move around within the hospital and come-and-go from the hospital (Table 1). All of the hospitals where the nurses worked prohibited any visitors during the postpartum stay aside from a single support person. As the pandemic waxed and waned in local communities, hospitals loosened or tightened visitation policies accordingly. Some hospitals allowed support persons to switch out at specified intervals (e.g., 12 or 24 h). For instance, the father could be present the first day and then go home and the grandmother come to stay for the second day. Policy also varied across hospitals and over time regarding whether support persons were permitted to leave the hospital and return during the mother’s stay. In most cases where the mother was not positive for COVID-19, support persons were permitted to leave the room to get food from the cafeteria or get items from their car. There was greater variation as to whether the support person could leave the hospital premises and return. In later months of the pandemic, many hospitals allowed the support person to go home to collect belongings or get food to bring back to the hospital; others did not. Some allowed a support person to leave the hospital, but they could not return until the next day. One of the hospitals in this study eventually began permitting two support persons during the mother’s postpartum stay.

**Table 1:** Changes in hospital policy relative to support person and family involvement during COVID-19

Sub-Categories	Quotations
Support Person Policy	“When we first started out, we were allowing no visitors except for dad. And that was at the very beginning where we kind of shut if off to anybody but dad. And that was a struggle for moms who wanted their birth doulas or their photographers or their mother or families.”
Come-and-Go Policy	“So they can stay, yeah, just that one person. And they get the band and they can stay, they can’t come and go. Like go home for the night and come back the next day, they have to stay the whole time. And our cafeteria accommodates them with a guest tray..”
Support Person Accommodations	“Dads can be there. They can come and go [for non-COVID positive patients]. And then they have sofas like a twin bed that we have for them. Its just not the ideal comfortable.”

The nurse participants noted that in most cases, the support person (usually the father), stayed with the mother for the entire stay. Hospital accommodations for support persons included either a couch or pull-out bed, and in some cases, queen size beds that they could share with the mother. If support persons were not permitted to leave the hospital, they were either provided with a tray of food like the mother, or they could purchase their own food in the cafeteria.

### Challenges for patient care and education

Nurses indicated that for patients who tested negative for COVID-19, they visited patient rooms and carried out all their usual care and education activities, including lactation support, in the same way and to the same degree as they had prior to the pandemic. Nevertheless, social distancing measures taken by hospitals reduced opportunities for hands-on teaching and learning in some areas. For example, one nurse noted that her hospital cancelled their childbirth and parenting classes previously offered in-person or switched them to a virtual format. Nurses found the virtual format to be somewhat less effective in conveying information that involves hands-on tasks, such as diapering, bathing, and swaddling a newborn. They also felt that a virtual format, particularly when video was not available, made it more difficult for them to gauge parents’ level of understanding of the material. See Table 2 for example quotations. It was noted by many nurses that because classes had been cancelled, parents were arriving at the hospital for their delivery less prepared than they were prior to the pandemic. Further, some in-hospital policies even exacerbated this lack of preparedness. In some hospitals, parents were not able to participate directly in the baby’s first bath because they were barred from the nursery due to social distancing rules. In another hospital, infants were wheeled over to the door, away from the mother’s bedside, for assessments, tests, and baths. These distancing measures may have limited opportunities for teaching and learning during these important activities.

**Table 2:** Perceived challenges for patient care and education due to COVID-19

Sub-Categories	Quotations
Less Hands-on Parent Education	“Unfortunately, when it comes to our online classes, I’m not able to have their video cameras on… so now I just see a brief name, I can’t see them anymore. So I’m not sure who’s attending. So it’s a little more difficult for me to... I don’t feel like I’m getting everything that I want to get across to everyone.”
	“There are things that we had in place that I liked that we have kind of stopped doing education wise, like car seat education and stuff…. We had like a car seat that we would take into the rooms and it had a baby in it and we would like show them how to proper installation of a car seat and making sure baby’s in there safe. But we don’t necessarily like bring that car seat in and out now.”
	“Prior to COVID, I really liked to bring both parents into the nursery… for the first bath, especially first-time parents and let them really be involved in that process so they could feel comfortable and not be reluctant when that time came at home. Unfortunately, that’s something that we’re no longer able to do. We can’t take families into the nurseries…. And so that has been sort of a downside because then they’re just kind of watching through the window.”
Early Discharge	“… So you have the patient asking, but you also have the physicians offering to send them home sooner. It just kind of speeds it up with how much time you have to give them teaching and how much time they have in the hospital to receive help from lactation or stuff like that.”
	“And so just [from a] nursing standpoint, there’s just a lot to do, a lot that has to be done in 24 h for mom. We want to make sure pain’s under control before we send her home. And then baby, the hearing screens, CCHD, the PKU, make sure [bilirubin] is okay. So there’s a lot of things that has to be done, and sometimes it can be rushed. Well, as a nurse, I feel rushed...”
	“[Early discharges] affects [my ability to do my job] a lot because we have less time. We try to get all the tests done and still ensure the safety and provide all the care that we’re supposed to, to those patients in less timeframe…. I do feel a little rushed when they request … to go home early.”

Additionally, nurses reported that many families opted for early discharge during the pandemic, which meant nurses had significantly less time to perform their many duties. A typical postpartum stay prior to the start of the pandemic was approximately 2 days for a vaginal birth without complications, and up to 4 days for a caesarean birth. During the pandemic, nurses reported that many families opted to leave after 24 h for a vaginal birth, and 48 h after a caesarean (assuming there were no complications). Nurses perceived various reasons for the increase in early discharges, including parents’ fear of COVID-19 infection, feeling isolated from family and friends, lack of childcare for older siblings, and feeling “cooped up” or “trapped” in the hospital or room.

While early discharge alleviated some of the emotional and logistical strains facing parents during the pandemic, it significantly limited the amount of time nurses had to provide care and education to parents. Nurses reported that they “try to get all the tests and still ensure the safety and provide all the care that we’re supposed to, to those patients” in a shorter timeframe, and as a result had felt “rushed.”

### Benefits for patient care and education

Despite these challenges, nurses’ attitudes toward COVID-19-related policy changes for patients who were not COVID-19 infected -- specifically restrictions on visitation -- were overwhelmingly positive (see Table 3). Nurses reported that they were able to spend more time interacting with parents one-on-one and that these interactions were more focused and productive because nurses could give the parents their undivided attention, and vice versa. Additionally, participants felt strongly that these more focused interactions led to more effective education. They indicated that they were better able to communicate information to parents when it was just the three of them in the room. Some also reported that parents were more interested and able to ask questions and focus on the information the nurses were presenting. An additional benefit mentioned by nurses was that they found it easier to teach parents best practices in newborn care (e.g. placing infants on their back to sleep) without conflicting opinions or information coming from other family members. They also felt it was easier to correct parents when they were alone, as they were hesitant to “call out” parents’ mistakes in front of family and friends.

**Table 3:** Perceived benefits of visitor restrictions for patient care and education during COVID-19

Sub-Categories	Quotations
More nurse-parent interaction	“I definitely have more time to interact with my patient and… with the dad. Because every time I come into the room, if there’s visitors, other than dad, then I usually just check on them real quick and leave the room, but if there’s only mom and dad and baby, I can have a little more conversation and answer more questions. Yeah, definitely spend more time with mom and dad during the COVID.”
	“I think that there’s been a tremendous benefit and it being limited to just mom, dad, and baby, we’re able to really focus in on what they need. And they’re able to have that space to ask questions that they might not have otherwise asked if family and visitors were in the room.”
	“I think it’s better. Like I said, not having to deal with visitors coming in and out; we got more time for each mom and dad. I get to focus more on them and get to spend more time with them.”
More effective education	“… I’m able to not just teach them, but also get to know them and their family dynamic at home, whether it be them having older kids, and how to get them involved, and just how mom and dad interact with each other, and how they can help each other out.”
	“Like I enjoy, I mean, not having all those visitors because… you couldn’t really interact with the mom and dad because they were too busy trying to entertain everybody and you couldn’t really teach them. But now with nobody in there, they’re very involved and they want to learn…”
	“So I feel like it has been better, in a sense, that it’s kind of more one on one and not so mom, grandma, grandpa, and cousins are in the room. So giving education may not be spread between eight people, and not really direct. So that’s been a little bit easier, it’s more one on one.”
More father involvement	“Without those outside influences… in some ways maybe it gives dad a little bit more space to be there, and ask his own questions, and… there’s less pressure from their mother-in-law, or the mom’s mom, I guess.”
	“I think them just being there more has increased their involvement. So the visitation policy changing has had that side effect of dad is pretty much restricted to the room, they’re not even out walking in the hallways… So yeah, just the policies of them being in the room has lent itself to us having a lot more time to educate and involve them.”
	“Now, since COVID, dads are the only support people there, we don’t have all these extra family members and friends trying to also be in the postpartum room right away. And so usually when I’m doing my first assessment….I’m able to… demonstrate that first diaper change and kind of talk through my assessment and show him what’s normal as well. There’s just a lot more time and space for that now I feel.”

The absence of other visitors besides the support person, who was usually the father, also allowed nurses to provide more targeted attention and education to fathers and to involve them more in newborn care.^1^ Because support persons were often restricted from leaving the room or the hospital, nurses reported that they had more facetime with fathers than before the pandemic. Most perceived that fathers were more engaged in all aspects of postpartum care and education, largely because they were the only person there to help. Some felt that the more intimate setting opened up more opportunities for fathers to ask questions, share opinions, and learn to care for their partner and newborn without interference from others.

### Improved patient outcomes

Nurses not only perceived benefits for their own job performance because of restricted visitation, but also for patient and family outcomes. Among the benefits commonly cited were more maternal rest, more breastfeeding, and more family bonding time (see Table 4). One participant noted that infants in her unit experienced less weight loss after birth, because mothers were able to focus more on newborns’ hunger cues and to feed more frequently. Participants also noted that greater privacy led to greater intimacy and more time for parents to process their experience, adjust to their new infant, and bond as a family unit.

**Table 4:** Perceived improved patient and family outcomes related to visitor restrictions during COVID-19

Sub-Categories	Quotations
More Maternal Rest	“I feel like our moms are much more well rested since we don’t have visitors. I feel like they are breastfeeding better. I feel like bonding and stuff is better and there’s less stress for mom to have to worry about constant visitors in and out of the hospital and not worrying about breastfeeding in front of her family and that kind of stuff.”
More Breastfeeding	“The lack of visitors too has helped with breastfeeding. It’s helped with both of the parents getting more rest because they’re not worried about people coming in…”
More Family Bonding Time	“We find that moms are resting better, which helps with their blood pressure…. We noticed that moms are breastfeeding better. They’re sleeping better. They’re asking questions. They’re learning stuff. Dad is getting involved. Dad does skin to skin... It’s bonding time for mom, dad, and baby, or mom and baby.”

One participant noted that infants in her unit experienced less weight loss after birth, because mothers were able to focus more on newborns’ hunger cues and to feed more frequently. Participants also noted that greater privacy led to greater intimacy and more time for parents to process their experience, adjust to their new infant, and bond as a family unit. One participant summarized the multiple benefits of restricted visitation saying: “We find that moms are resting better, which helps with their blood pressure…. We noticed that moms are breastfeeding better. They’re sleeping better. They’re asking questions. They’re learning stuff. Dad is getting involved. Dad does skin to skin... It’s bonding time for mom, dad, and baby, or mom and baby.”

## Discussion

The public health demands of the COVID-19 pandemic imposed extraordinary constraints on family and social life and curtailed individual freedoms in various ways. Healthcare institutions must weigh the costs and benefits of social distancing and other restrictions as circumstances and priorities continue to shift. The findings of this study raise important questions about restrictions on support persons and visitors in family-centered postpartum settings.

Nurses in this study held overwhelmingly positive views on restricted visitation policies instituted in their hospitals during COVID-19. According to their self-report, the absence of visitors provided the temporal, physical and mental space for nurses, patients, and the support person to experience the types of interactions that exemplify quality FCC (see Buchko, Gutshall and Jordan [11] and Weiss and Lokken [12]). Nurses also believed that restrictions on visitation produced positive experiences and better outcomes for patients and their families. While this study did not include patient interviews, and therefore cannot verify nurses’ beliefs, studies of postpartum women’s learning needs and preferences have found that mothers value one-on-one instruction and time spent with their nurse, saying that it empowers them to better care for themselves and their newborn at home [11, 25]. They have also expressed the desire for greater attention and support for fathers, and for mothers and fathers to be treated as individuals, with distinct needs for learning and support [13, 20, 25]. Our findings suggest that restricted visitation in postpartum units may give nurses the space and time to provide higher quality, family-centered care. Additionally, nurses’ perceptions that parents were enjoyed the extra rest and family bonding time is validated by research that shows that mothers do, in fact, value “quiet time” without visitors, to allow them better rest and recuperation as well as private family time [25, 26].

However, it is also possible that nurses’ perceptions of improved care and education are overstated in comparison with patient and support person experiences, as was found in Bradfield et al. [19]. Other research has shown that patients and support persons highly value access to social support during the postpartum stay. In a study by Gaboury, Capaday, Somera, and Purden [25], both mothers and fathers expressed the desire for visitation policies that allow visitors (family or otherwise) at flexible times during their stay. Nurse perceptions of patient and support person experiences were significantly more positive than was found by Vasilevski et al. [20]. Vasilevski [20] found that both mothers and their support persons experienced distress and dissatisfaction with their postpartum experience during COVID-19, noting that they felt hospital staff were stressed and overworked, that they did not receive enough support during their visit, and that they felt unprepared to take the baby home.

### Limitations

There are certain limitations inherent in the qualitative approach to research that should be noted here. Readers must keep in mind that findings of the present study reflect nurses’ *perceptions* of changes in patient care, education, and patient/family experience under COVID-19 visitor restriction policies. Alternative research methods, such as observational and/or quantitative measurement are necessary to assess actual differences in the quality and effectiveness of nurse-patient communication, education, and father involvement under restricted visitation. Additionally, the present study methodology is not adequate to establish whether any changes in these outcomes are causally linked with restricted visitation; randomized studies contrasting outcomes between a group with restricted visitation and one with unlimited visitation are needed to assess causal relationships.

### Implications for policy and practice

Study participants reported that, prior to the start of the pandemic, their hospitals had no restrictions on visitation. Findings of this study suggest that unlimited visitation may pose challenges both for nurses who are trying to provide care and education during this brief but critical time, and for families who are trying to incorporate a large amount of information in a short time span while bonding with their new baby. Many nurses in the study said they hoped restrictions would continue after the pandemic, and one said her director was already planning to retain some of the COVID-era visitation policies in the long term, because of the observed benefits to patients and their families.

Family-centered postpartum units may wish to restore policies that favor patient and family access to their infants, patient and support person autonomy (e.g., mobility), and provide opportunities for hands-on teaching and learning (perhaps with additional virtual options for those who prefer it). Findings of this investigation suggest that, rather than a wholesale return to unrestricted visitation, hospitals may wish to explore options that balance patients’ access to family and social support, with the benefits of rest, bonding time, and individualized education for the mother and support person(s).

However, more research is needed before specific policies can be formulated or recommended. Studies exploring patients’ preferences for postpartum visitation policies, as well as impacts of different visitation models on patient satisfaction and health outcomes are needed. Nurses in this study perceived benefits of restricted visitation for patients including more rest, more breastfeeding and skin-to-skin care, and greater parent engagement in education and newborn care. Quantitative evidence is needed to verify these perceptions from more healthcare providers and importantly, from the families. Studies should not only assess impacts on health behaviors and outcomes, but on learning outcomes and parental competency and self-efficacy, as well. Additionally, research may identify a need to provide nurses with training and education to improve attitudes and practices toward FCC. Studies conducted in postpartum and other healthcare contexts have found that nurses often hold attitudes inconsistent with and experience challenges implementing family-centered models of care [27–33].

## Conclusion

Family-centered models of care place the needs and wishes of the patient and family at the forefront. Many hospitals have interpreted and implemented the FCC principle of access to family and social support through policies of unlimited visitation during the postpartum stay. However, present findings suggests that some limitations on visitation may achieve important, family-centered goals. Protected time for family-bonding, maternal rest, breastfeeding, father involvement and individualized education are critical to quality FCC. Research must examine which visitation policies maximize these benefits while balancing patient access to family and social support.

## Data Availability

The datasets generated and/or analysed during the current study are not publicly available because they contain personally identifiable information; data are available from the corresponding author on reasonable request.

## Abbreviations

COVID-19: Coronavirus Disease of 2019
FCC: Family-centered Care
NICU: Neonatal Intensive Care Unit
QD: Qualitative Descriptive

## Footnotes

1 In this paragraph, and in Table 1, references are to “fathers” rather than “support person” because nurses were asked to reflect on father engagement, specifically, as part of the larger study of nurse-father interaction.
